# ‘From rhetoric to action: Moving policy, research, and practice’ – The 29^th^ Canadian Conference on Global Health in 2023

**DOI:** 10.7189/jogh.14.02001

**Published:** 2024-12-13

**Authors:** Michelle Amri, Margaret J Mutumba-Nakalembe, Johanna C Manga, Colleen M Davison

**Affiliations:** 1 The W. Maurice Young Centre for Applied Ethics, School of Population and Public Health, University of British Columbia, Vancouver, British Columbia, Canada; 2 Frugal Biomedical Innovations, School of Biomedical Engineering, Western University, London, Ontario, Canada; 3 Canadian Association for Global Health, Ottawa, Ontario, Canada; ^4^Department of Public Health Sciences, Queen's University, Kingston, Ontario, Canada

The Canadian Conference on Global Health (CCGH) is an annual conference organised by the Canadian Association for Global Health that brings together researchers, practitioners, policymakers, and students from various disciplines and sectors to discuss pressing global health issues. The CCGH provides a platform for sharing knowledge, best practices, and innovations in global health research, policy, and practice.

The 29^th^ CCGH, co-chaired by Drs Michelle Amri and Margaret Mutumba, was held in person from 16 to 18 October 2023 in Ottawa and virtually [1]. It featured over 120 speakers and 1319 registered delegates from across 39 countries and created a broad platform for experts, health and healthcare professionals, policymakers, researchers, and students to engage, share perspectives, and explore current and emerging topics within the sphere of global health.

The theme for the 29^th^ CCGH, ‘From rhetoric to action: moving policy, research, and practice’, invited participants to consider how rhetoric in global health can be turned into action, whether that be in terms of program and policy implementation, practice, or advocacy, to deliver positive results. The nature of global health is arguably centred on health equity, inclusivity, and research and practice. Global health addresses transnational health issues, is multidisciplinary, and takes a multisectoral approach. Each of these aspects, in addition to other aspects inherent to global health, have been studied, considered, and discussed to varying degrees. For example, there are numerous epidemiological studies mapping the prevalence of HIV, tuberculosis, malaria, and so forth. Prioritising continued publication and discussion of such issues, although valuable, may not always translate into practice. Now what is often missing is an emphasis on moving from rhetoric to action. What do we need to do next to take the knowledge we have to advance policy, research, and practice in light of resource constraints and lack of political will? We are at an opportune time to consider how we can act in our roles as scientists, practitioners, clinicians, advocates, citizens, and many others, to deliver evidence-informed and tangible outcomes. Thus, the 29^th^ CCGH considered how we can turn rhetoric in global health – whether that be in terms of programme and policy implementation, practice, or advocacy – into action to deliver positive results to the beneficiaries of global health efforts. Under this broader theme, the conference also featured four sub-themes, which are detailed below.

## SUB-THEME 1: HOW DO WE ENSURE NO ONE IS LEFT BEHIND?

This sub-theme focussed on striving for equality, health equity, and reducing health inequities everywhere. We took a holistic approach to this sub-theme, meaning that it was focussed on both health equity and health inequity directly, and indirectly, including focussing on designated groups. There were no restrictions on groups of interest for this sub-theme, as we wanted to learn more about action directed to individuals and groups affected by conflict and structural violence, environmental, social, political, and historical factors, and intersections thereof. We received submissions that considered how health equity or inequity, and challenges related to health inequity, can be or have been acted on to improve health outcomes for groups.

## SUB-THEME 2: FOSTERING PARTNERSHIPS WITH SHARED BENEFITS IN GLOBAL HEALTH

This sub-theme focussed on creating shared benefits, one of the six Principles for Global Health Research developed by the Canadian Coalition for Global Health Research (one of the predecessor organisations to the Canadian Association for Global Health) [2]. Creating shared benefits in global health entails considering the impacts, distribution, and sustained benefits of research, training, and practice. We invited submissions that considered the tangible outputs and outcomes of how groups have or can benefit from research, training, and practice through mutually beneficial partnerships.

## SUB-THEME 3: HOW ARE INNOVATION, TECHNOLOGY, AND DIGITAL HEALTH TRANSFORMING GLOBAL HEALTH?

This sub-theme focussed on how innovation, technology, and digital health are transforming global health. The emergence of new technologies affords new opportunities and ways of acting on rhetoric in global health; in addition to existing technologies (e.g., social media) that lead to mis- and dis-information. We invited submissions looking at how innovation, technology, and/or digital health can and have transformed global health research and practice, including advocacy and awareness.

## SUB-THEME 4: UNPACKING THE ROLE OF POLICY AND POLITICS IN GLOBAL HEALTH

Global health is influenced by political determinants of health. How we conduct our research and practice, how we identify the population(s) of interest, how we conduct evaluations, how research and practice are communicated, how we advocate for more equitable health and well-being, and how global health is funded, are just a few examples of the political nature of global health. We invited submissions that interrogated the political dimensions of global health, how policy in global health can be political, and/or global health diplomacy, with discussions of potential next steps for action, moving beyond tools, approaches, and global actions that are already in place.

## CONCLUSIONS

Following the 29^th^ CCGH, the Canadian Association for Global Health surveyed all attendees, speakers, and sponsors. The survey results highlighted the conference’s substantial role in augmenting knowledge of the latest global health research and in boosting the capacity to develop interventions targeting power dynamics, including gender, and promoting equity among attendees. More specifically, in-person attendees praised the diversity in themes and the global representation of speakers. Further, the commitment to bilingualism was particularly strong, as demonstrated by the three fully bilingual moderators across the four plenaries and the inclusion of seven francophone plenary speakers, enhancing the diversity and inclusivity of discussions.

Looking ahead, the 30^th^ CCGH in 2024 was held in person from 25 to 27 October 2024 at Simon Fraser University’s downtown Vancouver campus and virtually [3]. This year’s theme was ‘Poly-crisis and global health: How can we improve human health and equity while protecting the planet?’. We invited colleagues to join us – whether in-person or virtually – as we addressed the drivers and immediate impacts of poly-crisis and laid the foundation for ongoing strategies to mitigate and adapt to the poly-crisis to ensure more equitable, inclusive, resilient, and sustainable global health ecosystems. We emphasised equity, social justice, community engagement, proactive involvement of population groups in situations of vulnerability and marginalisation, and authentic partnerships between researchers and practitioners from the global south and global north.
